# Reproductive issues in patients undergoing Hematopoietic Stem Cell Transplantation: an update

**DOI:** 10.1186/s13048-016-0279-y

**Published:** 2016-11-01

**Authors:** Maurizio Guida, Maria Antonietta Castaldi, Rosa Rosamilio, Valentina Giudice, Francesco Orio, Carmine Selleri

**Affiliations:** 1 Department of Medicine and Surgery, University of Salerno, Salerno, Italy; 2 Ph. D. Program in Translational Medicine, Department of Experimental Medicine, Second University of Naples, Naples, Italy; 3 Department of Maternal and Child Health, Operative Unit of Obstetrics and Gynaecology, A.O.R.N. S.G. Moscati, Contrada Amoretta, 83100 Avellino, Italy; 4 Department of Sports Science and Wellness, “Parthenope” University of Naples, 80133 Naples, Italy

## Abstract

In 1963 George Mathé announced to the world that he had cured a patient of leukaemia by means of a bone-marrow transplant. Since than much progress has been made and nowadays Hematopoietic Stem Cell Transplantation (HSCT) is considered the most effective treatment of numerous severe haematological diseases. Gynaecological complications in HSCT women represent a serious concern for these patients, but often underestimated by clinicians in the view of Overall Survival. The main gynaecological complications of HSCT are represented by: premature ovarian failure (POF), thrombocytopenia-associated menorrhagia, genital symptoms or sexual problems in course of chronic GVHD (cGVHD), osteoporosis, secondary solid tumours due to immunosuppressive drugs to treat cGVHD and severity of cGVHD, and fertility and pregnancy issues. In particular fertility-related issues are always more relevant for patients, whose life expectation is constantly growing up after HSCT.

Thus, taking care of a patient undergoing HSCT should primarily include gynaecological evaluation, even before conditioning regimen or chemotherapy for the underlying malignancy, as, in our opinion, it is of great importance to ensure a complete diagnostic work-up and intervention options to guarantee maximum reproductive health and a better quality of life in HSCT women.

The present review aims at describing principal features of the aforementioned gynaecological complications of HSCT, and to define, on the basis of current international literature, a specific protocol for the prevention, diagnosis, management and follow-up of gynaecological complications of both autologous and heterologous transplantation, before and after the procedure.

## Background

In 1963 George Mathé announced to the world that he had cured a patient of leukaemia by means of a bone-marrow transplant [1].

Since than much progress has been made and nowadays Hematopoietic Stem Cell Transplantation (HSCT) is considered the most effective treatment of numerous severe haematological diseases [2]. The most common sources of multipotent hematopoietic stem cells are bone marrow, peripheral blood or umbilical cord blood. Autologous transplantation – autograft or auto-HSCT– (hematopoietic cell transplantation in the same patient after appropriate treatment) differs from allogeneic transplantation (allograft or allo-HSCT), in which the marrow comes from a donor [2, 3].

Allo-HSCT is associated with a more severe derangement of the immune system than auto-HSCT. Main factors with important effects on post-transplantation period are: conditioning regimens administered to avoid graft rejection, cytokine storm triggered by transplantation, multiple immunosuppressive therapy to prevent graft-versus-host disease, and frequent development of acute/chronic multi-organ Graft Versus Host Disease (GVHD) [4].

Gynaecological issues before and after HSCT have been under investigation since the early 90s [5, 6]. Actually the main gynaecological complications of HSCT are represented by: premature ovarian failure (POF), thrombocytopenia-associated menorrhagia, genital symptoms or sexual problems in course of chronic Graft Versus Host Disease (cGVHD), osteoporosis, secondary solid tumours due to immunosuppressive drugs to treat cGVHD and severity of cGVHD [7, 8], and fertility and pregnancy issues. Nonetheless, these last issues remain poorly investigated in the international literature with only some reports of successful pregnancies [9].

Therefore, fertility preservation in women undergoing HSCT should represent a major concern for the clinician taking care of these patients, who have a risk higher than 80 % of permanent amenorrhea after HSCT with cyclophosphamide/total body irradiation (TBI) or cyclophosphamide/busulfan [5, 10–12].

The first part of the present review aims at describing principal features of the aforementioned main gynaecological complications of HSCT.

Otherwise, in the second part, which represents the aim of the present paper, we define, on the basis of current international literature, a specific protocol for the prevention, diagnosis, management and follow-up of gynaecological complications of both autologous and heterologous transplantation, before and after the procedure.

## Methods

This review includes papers published in the English language on Hematopoietic Stem Cell Transplantation and Gynaecological complications since 1997 and identified through a MEDLINE and EMBASE search using combinations of the following subject heading terms: Hematopoietic Stem Cell Transplantation, gynaecological complications, cGVHD, fertility, fertility preservation, reproduction. Of the retrieved articles, 73 papers regarding gynaecological complications of HSCT were added, together with guidelines of different societies and international recommendations from experts were added. Finally, our specific protocol for the prevention, diagnosis and treatment of gynaecological complications before and after HSCT women is described.

### Gynaecological complications of HSCT

#### POF

POF, also known as ovarian insufficiency, is characterized by loss of normal ovarian function before the age of 40 and usually occurs as secondary amenorrhea associated with hypergonadotropic hypogonadism, and reduced volumes of ovaries and uterus [4].

POF is characterized by symptoms of oestrogen deficiency, such as hot flushes, night sweats, mood disorders, sleep disorders, lack of concentration, arthralgia, impaired sexual function and loss of reproductive potential, that have a negative impact on quality of life [4, 7].

Lack of oestrogens at an early age prevents the development of secondary sex characteristics, increases the risk for osteoporosis, cognitive impairment and cardiovascular disease, frequently observed in women after HSCT [7, 13].

POF is the most frequent complication after stem cell transplantation for haematological malignancies, occurring in more than 90 % of women [14, 15]. Indeed, conditioning regimens consisting of high-dose antiblastic treatments associated or not with TBI, precede both auto- and allo-HSCT procedures, causing gonadal damage [4]. Moreover, allo-HSCT can be followed by acute or chronic GVHD and long-lasting steroid treatment, which can further increase ovarian damage [4]. Risk factors for gonadal damage in women undergoing HSCT are summarized in Table 1.

**Table 1:** Risk factors for gonadal damage in women undergoing HSCT^a^

Patient relating factor	
Pubertal stage	Postpuberal > prepuberal
Age of HSCT	>30 years
Underlying disease	ALL, lymphomas
Treatment relating factor	
Type of radiotherapy	TBI, pelvic, inverted Y, fractionated doses
Chemotherapy	Alkylating agents > other chemotherapy
Type of transplant	Allo-HSCT > auto-HSCT
HSCT complications	Presence of GVHD

*ALL* acute lymphoblastic leukaemia, *allo* allogeneic transplantation, *auto* autologous transplatation, *GVHD* graft-versus-host disease, *HSCT* hematopoietic stem cell transplant, *TBI* “total body” irradiation

Symbol (>) means more than

^a^ modified from Orio et al. [4]

#### Thrombocytopenia-associated menorrhagia

Menorrhagia is defined as heavy regular menstrual cycles with more than 80 ml of blood loss/cycle or cycle duration longer than 7 days.

After different conditioning regimens, women in reproductive age may experience thrombocytopenia-associated menorrhagia, especially if they had experienced regular or irregular vaginal bleeding before HSCT [16]. This condition is a serious complication in young female oncology patients who suffer from severe thrombocytopenia during myelosuppressive treatment, associated with significant morbidity. Consequently, effective management is necessary to prevent adverse outcomes [17].

Actually, in order to manage thrombocytopenia-associated menorrhagia, physicians can use the Menorrhagia Impact Questionnaire (MIQ) [17], developed to identify measurement domains, define meaningful, symptom relief, measure magnitude of response, assess a patient’s ability to perform physical functions, work, and participate in social and leisure activities, as well as measures global change in menstrual blood loss.

Gonadotropin-releasing hormone analogues (e.g., leuprolide) are promising therapies that have been shown to decrease vaginal bleeding during periods of thrombocytopenia and to have minimal adverse effects other than those associated with gonadal inhibition [16, 17]. In patients who experience menorrhagia despite preventive therapies, or in patients who have thrombocytopenia and menorrhagia at diagnosis, treatment is indicated. For these women, treatment options may include platelet transfusions, antifibrinolytic therapy (e.g., tranexamic acid), continuous high-dose oral contraceptives, cyclic progestins, or other therapies for more refractory patients such as danazol, desmopressin, and recombinant factor VIIa. Hormonal therapies are often the mainstay of therapy in women with menorrhagia secondary to thrombocytopenia, but data for these agents, such as combined hormonal contraceptive pills, combined hormonal contraceptive patch, depot medroxyprogesterone, and oral norethindrone are sparse [16, 17]. The most robust data for the treatment of menorrhagia are for tranexamic acid [16, 17].

#### Genital symptoms of cGVHD

Chronic GVHD is a serious and common complication of allogeneic hematopoietic cell transplantation (HCT), occurring in 30 % to 70 % of patients [18]. Chronic GVHD is a syndrome of variable clinical features resembling autoimmune and other immunologic disorders, with a pathophysiology involving inflammation, cell-mediated immunity, humoral immunity, and fibrosis [19, 20].

Chronic GVHD of female genital tract was firstly described in 1982: five cases of severe vaginal scarring successfully treated with surgery (to remove scar tissue) and use of oestrogen and vaginal dilators were described [21].

Pain, burning, dysuria, a feeling of redness and swelling, perineal and perianal soreness, dryness and dyspareunia, are nonspecific symptoms of female genital cGVHD. Patients, who are sexually active, may recognize symptoms earlier [19, 20].

Female genital cGVHD can cause complete vaginal obstruction resulting in haematocolpos or hematometra, which can manifest as cyclic pain and amenorrhea in women taking HT or as difficulty in voiding in case of vulvar adhesions [7]. Actually, female genital cGVHD is well-recognized clinical condition, with a precise assessment and scoring form recently developed [19].

Treatment options include: topical steroids, topical immunosuppressants (cyclosporine, tacrolimus), oestrogen therapy applied locally to vulva and vagina, and surgery if severe scarring is recognized [19].

#### Osteoporosis

Osteoporosis occurs when the creation of new bone does not keep up with the removal of old bone [22]. Significant effects on bone metabolism resulting from HSCT were observed [23, 24].

In particular, osteoporosis after HSCT is attributable to the influence of multiple factors including myeloablative conditioning regimens, huge cytokine re-lease at the time of transplant, altered kidney, liver and bowel function resulting in reduced intake and altered metabolism of calcium and vitamin D, in allogeneic HSCT setting, long-lasting high-dose steroids and cyclosporine-A [24]. Therefore, in women of reproductive age this is also associated with lack of oestrogens associated with secondary amenorrhea and gonadal failure due to the underlying illness with its complications and treatments [23, 24].

Moreover, bone marrow transplantation recipients are at risk of osteoporosis secondary to bone loss associated with their illness and/or chemotherapy, particularly in female autograft recipients, and in allograft recipients secondary to GVHD and its treatment [25].

Few data are available so far on the treatment of bone loss in HSCT recipients. Early diagnosis of osteoporosis or early bone senescence in this particular population is a major aim to promptly start appropriate supportive measures, such as lifestyle modification, calcium and vit-amin D supplementation or bisphosphonates [24]. Treatment of osteoporosis in women undergone HSCT actually include hormonal therapy (HT), which has been demonstrated to increase bone mineral density of the hypogonadal patients [23].

#### Secondary solid tumours

One of the long-term complications associated with allogeneic hematopoietic stem cell transplantation is the development of secondary malignancies [26–33]. Several factors contribute to the development of secondary neoplasm: the radiation and chemotherapy used for conditioning and for treatment of the primary malignancy, and the profound immunosuppression that occurs post transplant [26].

Indeed, the risk of new solid cancers after bone marrow transplantation in different studies resulted to be higher than in general population, in particular after allo-HSCT [26–30]. In particular, two large cohort studies published by the IBMTR and EBMT have confirmed the increased risk of secondary malignancy post transplant [27, 28].

These studies found that older age of female recipients or female donors, the number of immunosuppressive agents used for treatment of cGVHD, TBI with overall doses of 1000 cGy or higher, and the presence of cGVHD were significant risk factors for the development of secondary malignancy. The most frequent secondary solid tumours developed were: basal cancer cell skin [31], squamous cell cancer skin [32], squamous cell cancer oral [33] and breast cancer [32].

Moreover, prolonged use of immunosuppressive drugs to treat cGVHD and severity of cGVHD are risk factors for the development of human papillomavirus (HPV)-related dysplasia and squamous cell carcinoma of the female genital tract [34–36]. Thus, on one side, regular surveillance by gynaecologic examination, including cervical cytological testing, in these patients allows for early diagnosis and effective management of cervical abnormality and decreases the burden of this potentially fatal, but treatable, condition [35].

On the other side, the immunogenicity and efficacy of quadrivalent HPV vaccine has not been tested in long-term survivors after allo-HSCT [36].

#### Fertility and pregnancy issues

Fertility is clearly impaired after HSCT and the reported overall conception rate is <1 % [7]. Currently HSCT with cyclophosphamide/TBI or cyclophosphamide/busulfan leads to >80 % risk of permanent amenorrhea in women [5, 7].

Nowadays, people with cancer are interested in discussing fertility preservation. Health care providers, caring for adult and paediatric patients with cancer (including medical oncologists, radiation oncologists, gynaecologic oncologists, urologists, haematologists, paediatric oncologists, surgeons, and others), should address the possibility of infertility as early as possible before treatment starts [11].

In particular, patients undergoing HSCT, usually, have a restricted time-window to decide and undertake fertility preservation strategies. In this view, the classical oocytes/eggs freezing procedure could not represent the best option to be proposed to patients undergoing HSCT [5, 9]. Therefore, this procedure presents 2 main problems, as it includes at least a minimum of two-week-ovarian stimulation protocol. The first problem is the use of gonadotropins, which represent an open issue in cancer patients, and the second question is the two-weeks’ delay in the start of the conditioning regimen.

Actually, great effort is employing to improve both the communication between patients and physicians about fertility risks and preservation options, and the collaboration among haematologists and fertility specialists to give patients the opportunity to undergo well-timed and complete reproductive counselling [5].

Novel perspectives include strategies such as ovarian tissue freezing in young girls affected with various haematological diseases [5, 37]. This state-of-the-art procedure has been developed to preserve fertility in cancer patients.

However, not all the patients are considered eligible for cryopreservation strategies (i.e. due to need to start chemotherapy immediately [5]), so current clinical research is also focusing on agents which can prevent or attenuate the ovotoxic effects of treatment would provide significant advantages over the existing fertility preservation techniques, and would allow patients to retain their natural fertility without the necessity for costly, invasive and risky procedures [38].

### Ovarian tissue cryopreservation

Ovarian tissue cryopreservation is an investigational method of fertility preservation but has the advantage of requiring neither a sperm donor nor ovarian stimulation. Ovarian tissue is removed laparoscopically, a one hour outpatient procedure that requires general anaesthesia, and frozen. At a later date, the ovarian tissue is thawed and reimplanted. Primordial follicles can be cryopreserved with great efficiency [10, 39, 40], but because of the initial ischemia encountered after ovarian transplantation, a quarter or more of these follicles might be lost, as shown in xenografting studies [41]. To offset this relatively large loss, typically the cortex from an entire ovary could be cryopreserved in adults. The benefit of ovarian cryopreservation for women older than 40 years of age is very uncertain because there are too few primordial follicles remaining [42]. Ovarian tissue cryopreservation has been performed in humans for less than a decade, and the first ovarian transplant procedure was reported in 2000 [43], and the first pregnancy after ovarian transplantation in 2004 [44]. Ovarian tissue can be transplanted orthotopically to pelvis [44, 45] or heterotopically to subcutaneous areas such as the forearm or lower abdomen [46, 47]. Following re-implantation of ovarian tissue, a successful recovery of ovarian function is expected in almost all cases within 3 to 6 months, with possible sustained longevity of function of the transplanted tissue [48, 49].

To date, a total of 40 live births have been reported in cancer patients after transplantation of frozen/thawed ovarian tissue [50]. It is almost impossible to calculate a precise pregnancy rate for transplantation of cryopreserved ovarian tissue until a large cohort of patients has had all their tissue transplanted [51].

Combining 80 cases from 4 fertility centres, the pregnancy rate (i.e. ratio between the number of women who conceived and the number of transplanted women) with the use of this technique was 25 % [52–55]. Overall, the pregnancy rate with the use of cryopreservation of ovarian tissue seems to be increasing [5, 51].

One concern with reimplanting ovarian tissue is the potential for reintroduction of cancer cells. In patients without evidence of systemic metastasis to other organs, the likelihood of occult ovarian metastasis appears to be low in the majority of cancers seen in young females, and there are no reports of cancer recurrence after ovarian transplantation [56, 57]. Thus, ovarian tissue screening to detect malignant cells is currently performed to minimize the risk of inadvertent transfer with the ovary [9].

Ovarian cryopreservation and transplantation procedures should only be performed in centres with the necessary expertise under IRB-approved protocols that include follow-up for recurrent cancer, as this procedure has been proven to be an effective, yet still experimental, technique to preserve fertility in patients undergoing gonadotoxic therapies [5, 10, 11].

### GnRH analogues

Preserving patients’ natural fertility is the goal to which each reproductive health caregiver would lead. Recent data and reviews focus in particular on the use of GnRH analogues, also known as LHRH analogues, in fertility preservation of cancer patients undergoing cytotoxic treatments [38, 58–63].

LHRH analogues are used in various clinical situations, such as uterine fibroids, abnormal uterine bleeding, etc. LHRH analogues are widely used in oncology, and their mode of action is almost completely understood [58]. The mechanism of action of these analogues is mainly based on the inhibition of pituitary and gonadal function, but they also exert a direct function on the ovaries and uterus. Indeed, in germ cells, the cell cycle stage at the time of exposure will define the molecular response to cytotherapy [38, 57]. Between the primordial and antral follicle stages, the oocyte is in the dictyate (prolonged diplotene) stage of prophase I arrest [38]. Following the LH surge, the oocyte is triggered to enter into the first meiotic M-phase, M1 [64]. Thus, cytotoxic drug-induced apoptotic pathways have been well documented in growing follicles [65, 66]. On the other side, less is known on primordial follicles. A novel theory of chemotherapy-induced destruction of dormant follicles suggests that in vivo chemotherapy triggers follicle activation and growth, causing burnout and depletion of the ovarian reserve [67]. Histology demonstrated both an absence of apoptosis in primordial follicles together with an initiation of follicle growth immediately following chemotherapy exposure, occurring simultaneously with large follicle apoptosis [38].

Moreover, experimental data on mice show that intraovarian transplantation of primordial follicles fails to rescue chemotherapy-injured ovaries [68].

In this view, recent literature suggests that GnRH analogues could be protective of the ovarian reserve and natural fertility in cancer patients [38, 69]. The majority of clinical data comes from breast cancer patients, and only two papers report data on women with lymphoma [59, 70]. In particular, Triptorelin was not associated with a significant decreased risk of POF in young patients treated for lymphoma but may provide protection of the ovarian reserve [59]. Indeed, AMH levels were significantly higher in women who received cotreatment with GnRH analogue (1.4 compared with 0.56 ng/ml) [59].

However, despite this research effort, ovarian suppression with LHRH analogues during chemotherapy is still considered an experimental strategy to preserve fertility by some international guidelines due to both the uncertainty regarding the efficacy of this strategy and the absence of data on pregnancies and long-term ovarian function [11, 69].

### A clinic protocol for the management of gynaecological complications before and after HSCT

On the basis of the current literature and of our previous experience in the management of patients affected with different haematological diseases and/or treated with HSCT we define a unified protocol for gynaecological management in patients undergoing HSCT before and after the procedure.

#### Diagnostic work-up before HSCT

Taking care of a patient undergoing HSCT should primarily include gynaecological evaluation, even before conditioning regimen or chemotherapy for the underlying malignancy.

Before HSCT a general gynaecological evaluation should include: anthropometric characteristics, together with data on haematological conditions, serum ferritin levels. Data on menarche, including age and spontaneity of menarche and menses, are essentials when evaluating these women. Additionally, data on oestro-progestin (EP) replacement therapy, previous pregnancies (spontaneous or obtained with ovarian stimulation) and pelvic infections should be included in the work-up. Then, a gynaecological pelvic examination together with a sonographic scan, evaluating uterine size, endometrial thickness and ovarian size should be performed in each patient. In women of reproductive age, ovarian reserve should be evaluated including Antral Follicular Count (AFC) in the pelvic scan. Moreover, an hormonal evaluation together, an HPG function analysis, including basal levels of luteinizing hormone (LH), follicular-stimulating hormone (FSH), estradiol (E2), prolactin (PRL), anti-Mullerian hormone (AMH) and a thyroid profile (evaluation of blood levels of TSH, fT3, fT4, anti-TPO, anti-TBG) should be offered to all fertile women. This should be undertaken on day 2/3 of menses in women with a regular menstrual cycle, or on any random day in those with amenorrhea. Moreover, assessment of the partner, when possible, should be undertaken [5].

Finally, thromboembolic risk evaluation and bone density by MOC-DEXA of the vertebral district and of the right and left femur should be collected and eventually amended [15]. Women undergoing allo-HSCT should be assessed with the “Genital Tract Chronic Graft-versus-Host Assessment and Scoring Form” [11].

After that, an onco-fertility counselling should be offered to all patients undergoing HSCT. This counselling analyses type and dose of the chemotherapy regimen proposed, method of administration (oral versus intravenous), size and location of the radiation field and its dose, need of adjuvant therapy [10, 71]. Specifically, type of treatment and patients’ age are the most important factors to be taken into account when counselling the patients [72]. Onco-fertility counselling should be individualized, discussing both the absolute benefits of the proposed anticancer treatment (e.g. adjuvant chemotherapy) and the risk of infertility for each individual, proposing to each one the best treatment options. Gynaecological diagnostic work-up in patients undergoing HSCT is summarized in Table 2.

**Table 2:** Clinical and instrumental gynaecological first evaluation of women undergoing HSCT

Anthropometric Characteristics	• Age
	• Height
	• Weight
	• BMI
Haematological Data	• Kind of haematological malignancy
	• Kind, onset and duration of conditioning regimen
	• Serum Ferritin levels
	• Haemoglobin
Thrombotic Risk	• Previous thrombotic disease
	• PT, PTT, ATIII, fibrinogen
	• Platelet Count
Bone Evaluation	• MOC-DEXA of the vertebral district and of the right and left femur
Gynaecological Features	• Age at menarche
	• Spontaneous Menarche
	• Menses Regularity
	• Primary or Secondary Amenorrhea
	• Estro-progestins replacement therapy
	• Previous pregnancies
	• History of pelvic infections/inflammations
	• Gynaecological Examination
	• Pelvic ultrasound scan evaluating uterine size, endometrial thickness, ovarian size
	• HPV-test
Reproductive Features	• FSH, LH, estradiol (E2), prolactin (PRL), anti-mullherian hormone (AMH), and thyroid profile (TSH, fT3, fT4, anti-TPO, anti-TBG)
	• Antral Follicle Count (AFC)^a^
	• Onco-fertility counselling

^a^ During pelvic ultrasound scan

#### Gynaecological follow-up after HSCT

Gynaecological Follow-Up After HSCT should include a six-month complete evaluation including: collection of symptoms, pelvic scan and hormonal analysis of AMH, FSH, LH and estradiol. HPV-test should be repeated every six-month. Bone mineral density evaluation by MOC-DEXA of the vertebral district and of the right and left femur should be repeated every twelve months. Women underwent allo-HSCT should be assessed at each control with the “Genital Tract Chronic Graft-versus-Host Assessment and Scoring Form” [11].

#### Limitations

Gynaecological evaluation should start even before patients become candidates for HSCT, but early when they become diagnosed with a haematological malignancy. Indeed, even if the conditioning regimens are at higher dosage respect to conventional dosages, traditional chemotherapy however results in gynaecological complications [38]. Furthermore, current recommendations are not to allow patients to undertake cryopreservation strategies between chemotherapy treatments and to wait at least 6 months post chemotherapy before conception [32, 73].

## Conclusion

Gynaecological complications in HSCT women represent a serious concern for these patients, but often underestimated by clinicians in the view of Overall Survival. In particular fertility-related issues are always more relevant for patients, whose life expectation is constantly growing up after HSCT. Thus, in our opinion, it is of great importance to ensure a complete diagnostic work-up and intervention options to guarantee maximum reproductive health and a better quality of life in HSCT women.
